# Machine learning derived risk prediction of anorexia nervosa

**DOI:** 10.1186/s12920-016-0165-x

**Published:** 2016-01-20

**Authors:** Yiran Guo, Zhi Wei, Brendan J. Keating, The Genetic Consortium for Anorexia Nervosa, The Wellcome Trust Case Control Consortium 3, Price Foundation Collaborative Group, Hakon Hakonarson

**Affiliations:** 1 The Center for Applied Genomics, Abramson Research Center, The Children’s Hospital of Philadelphia, Philadelphia, PA 19104 USA; 2 Department of Computer Science, New Jersey Institute of Technology, Newark, NJ 07102 USA; 3 Department of Pediatrics, School of Medicine University of Pennsylvania, Philadelphia, PA 19104 USA

**Keywords:** Anorexia nervosa, Machine learning, Genome wide association, Risk prediction, Genotyping

## Abstract

**Background:**

Anorexia nervosa (AN) is a complex psychiatric disease with a moderate to strong genetic contribution. In addition to conventional genome wide association (GWA) studies, researchers have been using machine learning methods in conjunction with genomic data to predict risk of diseases in which genetics play an important role.

**Methods:**

In this study, we collected whole genome genotyping data on 3940 AN cases and 9266 controls from the Genetic Consortium for Anorexia Nervosa (GCAN), the Wellcome Trust Case Control Consortium 3 (WTCCC3), Price Foundation Collaborative Group and the Children’s Hospital of Philadelphia (CHOP), and applied machine learning methods for predicting AN disease risk. The prediction performance is measured by area under the receiver operating characteristic curve (AUC), indicating how well the model distinguishes cases from unaffected control subjects.

**Results:**

Logistic regression model with the lasso penalty technique generated an AUC of 0.693, while Support Vector Machines and Gradient Boosted Trees reached AUC’s of 0.691 and 0.623, respectively. Using different sample sizes, our results suggest that larger datasets are required to optimize the machine learning models and achieve higher AUC values.

**Conclusions:**

To our knowledge, this is the first attempt to assess AN risk based on genome wide genotype level data. Future integration of genomic, environmental and family-based information is likely to improve the AN risk evaluation process, eventually benefitting AN patients and families in the clinical setting.

**Electronic supplementary material:**

The online version of this article (doi:10.1186/s12920-016-0165-x) contains supplementary material, which is available to authorized users.

## Background

Anorexia nervosa (AN) is a complex eating disorder with psychiatric manifestations, including strong obsessive concern about gaining weight, twisted self-depiction towards body shape and eating, and extremely low food intake resulting in below-average body mass index [1–3]. The estimated prevalence of AN in the general population is ~ 1 % [4] and it is sex-biased, with an estimated female to male ratio of 10:1 [1, 3] and many patients being young women. Common comorbid psychiatric disorders include major depression disorder and anxiety disorders [5–9]. Among all psychiatric disorders, AN has one of the highest mortality rates [10–16]. However, interventions for AN have shown limited success and the hospitalization for weight regain is time consuming and expensive [17–19]. Altogether, AN incurs serious physical, psychological, familial and social toll to the modern world.

Recent efforts have shown that genetics plays an important role in AN susceptibility with heritability estimates from twin studies are as high as 84 % [20–26]. The inheritance is complex and multiple genes/loci are potentially involved, especially those in the dopamine pathway [27–29], weight/BMI related genes [30–33] and cholesterol metabolism regulatory pathway genes [34]. Three Genome Wide Association (GWA) studies [35–37] have been published without identifying an AN associated marker at genome-wide significance level of *P* value < 5E-8. Nevertheless, several genome wide marginal results have been reported, suggesting larger sample size and/or denser genotyping or high throughput parallel sequencing may be required to unveil the genetic underpinnings of AN.

Recently, machine learning based risk prediction methods using genotyping data have gained momentum in relation with GWA studies in complex disease [38–46], making an important contribution towards the promise of personalized medicine [47–49]. Here, we have organized the largest AN cohort so far [37], constructed machine learning models using GWA microarray data [47, 50], and applied the model to testing data set to evaluate the model’s performance by the area under the receiver operating characteristic curve (AUC) [47, 51, 52]. AUC is a value between 0.5 and 1 that assesses how well the model can distinguish cases from unaffected controls, with the higher number indicating better discriminative power. To our knowledge, this is the first attempt to assess AN disease risk using genetic information alone and the results, together with further investigation into AN genetics, would be anticipated to contribute to early diagnosis and allow for preventive interventions of AN in the future.

## Methods

### Ethics and consent

Local ethical approvals were granted for each participating study. For participants under the age of 16, written informed consent from their parents were obtained; for participants over the age of 16, written informed consent from themselves were obtained. Full names and locations of the ethic committees can be found in the end of the Additional file 1.

### Participating studies, genotyping and phenotyping

We combined individual level genotypic and phenotypic data from a total of 16 datasets in the Genetic Consortium for Anorexia Nervosa (GCAN)/Wellcome Trust Case Control Consortium 3 (WTCCC3), The Price Foundation Collaborative Group [53–55] and Children’s Hospital of Philadelphia (CHOP; Table 1). The final dataset encompassed 3940 cases and 9266 controls. Phenotyping details, genotyping approaches and quality control are described elsewhere previously [37]. In brief, all cases were female older than 9 years and met DSM-IV [1] (the Diagnostic and Statistical Manual of Mental Disorders, 4^th^ Edition) diagnostic criteria (amenorrhea criterion not required). Genotyping was done using Illumina microarrays, followed by quality control and imputation (as described previously in ref. [37]). We also collated 5087 disease free controls from Center for Applied Genomics at CHOP, which have been successfully used in previous neurodevelopmental/psychiatric GWA studies [56–59].

**Table 1:** Sample sizes of participating studies

Country	Cases	Controls
Canada	54	–
Czech Republic	72	–
Finland	131	404
France	293	–
Germany	475	–
Greece	70	–
Italy-North	203	–
Italy-South	75	–
Netherlands	348	–
Norway	82	–
Poland	175	–
Spain	186	–
Sweden	39	–
UK	213	–
USA	491	–
USA-CHOP	1033	8862
Total	3940	9266

### Logistic regression model

The entire dataset was randomly partitioned into three equal parts using Fisher-Yates permutation [60], without specifying case/control ratio. We then took a three step procedure to conduct the logistic regression (LR) prediction, including a) predictor pre-selection in the first subset of data (i.e. fold1), b) model training with cross-validation in the second subset (fold2) and c) model testing and assessment in the third part (fold3). As information for model training and testing were randomly drawn from the same collection of data, any population stratification in fold3 is already accounted for in the model during the training process in fold2 [61].

In the pre-selection stage, a GWA study was conducted in fold1 using PLINK [62] with conventional settings of maximal per-SNP missingness of 1 %, maximal per-individual missingness of 5 %, minimally allowed minor allele frequency of 1 %, minimally allowed Hardy-Weinberg equilibrium test *P* value of 1E-6. Then we retained SNPs with genome-wide case-control association test *P* value < 1E-3 to the next stage. Next we employed lasso regularized LR model with ten-fold cross validation in R package ‘glmnet’ [63] (http://cran.r-project.org/web/packages/glmnet/index.html) in fold2 data. A grid of lambda values (the regularization parameter in the model to reduce overfitting) are computed for the lasso penalty and AUC was measured to assess the performance. At the third stage, the model trained on fold2 data was then tested on fold3 data, and we calculated its AUC.

The procedure was repeated ten times using randomly shuffled datasets (Additional file 1: Table S1). Different sample sizes with randomized reruns were also examined to evaluate sample size effects to model fitting.

### Support Vector Machine and Gradient Boosted Trees

In order to compare different machine learning techniques, Support Vector Machines (SVM) with RBF kernels and default parameters (R package ‘e1071’; http://cran.r-project.org/web/packages/e1071/index.html) and Gradient Boosted Trees with default parameters (R package ‘gbm’; http://cran.r-project.org/web/packages/gbm/index.html) approaches were also used thereafter to train models within fold2 and assess the performance within fold3.

## Results

### Logistic regression model applied to the dataset

**Fig. 1:**
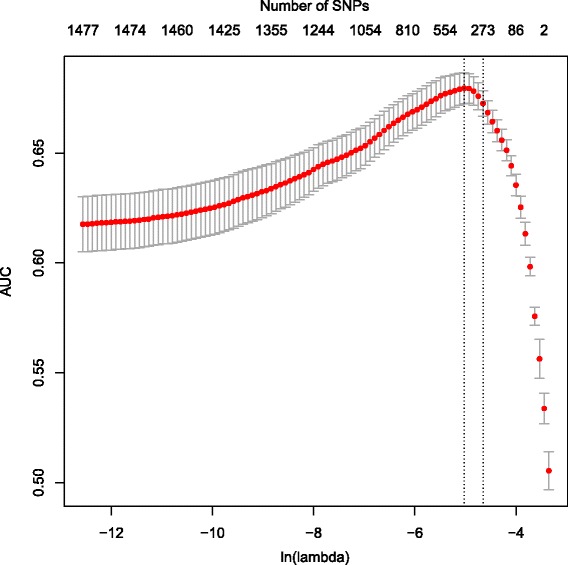
Logistic regression model with ten-fold validation. By harnessing L1 penalty (the lasso), we further removed irrelevant SNPs in fold2 after the preselection step in fold1. Smaller lambda (the penalty parameter) values correspond to fewer SNPs removed, and numbers on the top of the plot indicate how many SNPs survived with respect to specific lambdas as *X-axis* (natural logarithm scale). We estimated the mean and standard error (SE) for AUCs across 100 different lambda values, and reported the largest lambda such that AUC is within 1 SE of the optimum (the *left vertical dashed line* shows the lambda with maximum of AUC, while the *right vertical dashed line* shows the lambda with AUC being within 1 SE of that maximum). The optimal 10-fold cross-validated AUCs on fold 2 data was 0.673 with regularization parameter lambda of 0.00954

After combining 16 datasets (Table 1), imputed genotyping data were collated for a total of 3 940 cases and 9 266 controls each at up to 317 481 SNPs. We performed GWA scan in a subset of the cohort (the pre-selection dataset or fold1), which contains 1289 AN cases and 3113 healthy controls (Additional file 1: Table S1). Considering that currently no single marker has been found to be associated with AN at the level of *P* value < 5E-8 in any GWA study, we used SNPs with a less stringent threshold of *P* value < 1E-3, instead of the entire SNP list, for subsequent machine learning calculations. A total of 1486 SNPs were retained according to this criterion after quality control (Additional file 1: Table S1) and were utilized in LR model training and cross-validation. In the second subset of data with 1341 cases and 3061 controls (the model training dataset or fold2) where we did ten-fold cross-validation, the penalized LR with *L*_1_ penalty [63] (the lasso) generated a model of 273 SNPs (Additional file 1: Table S1), with an AUC of 0.673 and regularization penalty parameter (lambda) of 0.00954 (Fig. 1). Subsequently we fitted this model to the third subset of 1310 cases and 3092 controls (the testing dataset or fold3), and the result indicated an AUC of 0.693 (Additional file 1: Table S1, Fig. 1 and Additional file 2: Figure S1). We randomly shuffled the entire dataset nine more times and the results were similar for all runs (Additional file 1: Table S1). By using a 0.5 cutoff to the linearly calculated classifier output, we also calculated sensitivity and specificity in fold3, with values of 11 % and 97 %, respectively.

### Sample size effects to the model

**Fig. 2:**
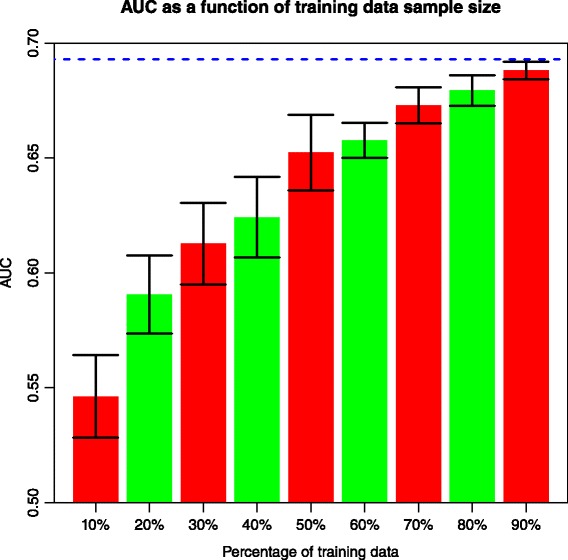
Relationship between smaller fold2 sample size (from 10 % to 90 % of the original) and AUC in fold3. 10 % of fold2 corresponds to 129 cases and 312 controls. Error bars with one standard deviation of 10 reruns are shown. *Blue horizontal dashed line* indicates AUC of fold3 when 100 % of the fold2 data were employed to train the LR model

**Fig. 3:**
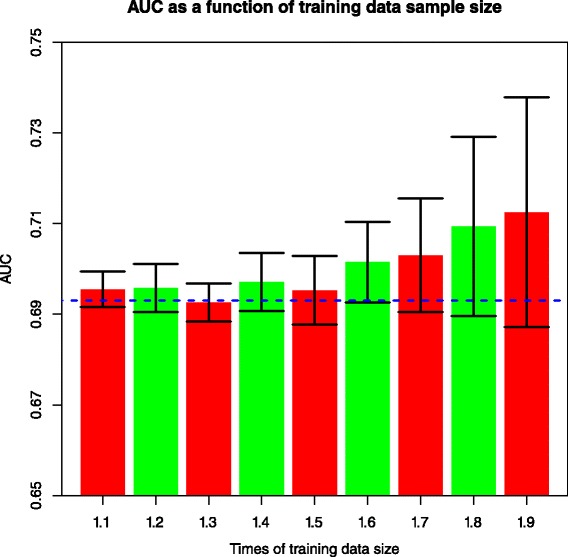
Relationship between training dataset with more samples and AUC in the model testing dataset, when moving samples from fold3 to fold2. 10 % of fold2 corresponds to 129 cases and 312 controls. Error bars with one standard deviation of 10 reruns are shown. *Blue horizontal dashed line* indicates AUC of fold3 when 100 % of the fold2 data were employed to train the LR model

We evaluated effects of sample sizes to the LR model performance. First, different percentages of fold2 samples were used for model training and the same fold3 dataset was fitted to measure the AUC values. We found a clear trend that AUC increases as the training population grows (Fig. 2 and Additional file 1: Table S2), which is consistent with the case of Inflammatory Bowel Disease (IBD) [44]. Results of ten randomized reruns showed significant differences between AUC’s of smaller training sample sizes and that of the original 100 % dataset (*P* values < 4.2E-3; Additional file 1: Table S2). We also experimented adding more samples from fold3 to fold2, in order to assess the behavior of the AUC trend in the situation of expanded training datasets. As shown in Fig. 3, AUC continues to increase along with the training dataset, especially when the training sample size is above 1.5 times of the original (*P* values < = 0.036; Additional file 1: Table S3), despite larger variation from ten randomized reruns.

### Comparison with other machine learning methods

We also explored two other widely used machine learning methods, Support Vector Machines (SVM) and Gradient Boosted Trees (GBT), and implemented them on our dataset following ten randomized repeats. While SVM provided similar performance in terms of AUC (*P* value = 0.099; Additional file 1: Table S1), GBT was significantly inferior to the LR method we used (*P* value = 6.9E-10; Additional file 1: Table S1).

## Discussion

We assessed AN disease risk using genome-wide SNP data by machine learning approaches on the largest AN cohort yet studied, representing one of the first applications of this kind in a complex psychiatric disorder [64, 65]. Our strategy follows a recent paper [66] with the basic idea that when data dimensionality is much larger than sample size, it is suggested to first do dimension reduction using a fast and simple method (e.g. univariate test), followed by some well-developed lower dimensional methods (e.g. lasso, Dantzig selector etc.). This can yield more accurate estimation as shown by our recent study [44]. After partitioning the cohort into three equal folds, we pre-selected SNPs in fold1 by filtering out those with genome-wide genetic association test *P* value > = 1E-3, trained the model in fold2 using LR with *L*_1_ penalty and cross-validation, and fit the model in fold3, achieving a discriminative measure AUC of 0.693. We used AUC mainly because this is a case-control study which makes it hard, if possible, to measure calibration accuracy. More discussion can be found in ref. [67] regarding model accuracy. With more experiments on sample size adjustment, we discovered that the AUC for AN risk prediction was similarly sample size sensitive as other complex disorders like IBD [44], suggesting that larger sample sizes are needed to improve the machine learning models. We also assessed the posterior probability generated with the LR model in fold3, and the sensitivity and specificity were 11 % and 97 %, respectively.

Our previous disease risk prediction efforts for type 1 diabetes (T1D) [39] and IBD [44] showed higher AUC as well as sensitivity/specificity values, which is consistent with, and can be explained by the fact that through successful GWA studies, genetic markers with significant association (e.g., *P* value < 5E-8) have been identified for both T1D and IBD, allowing for high performance machine learning models to discriminate cases from unaffected controls based on genotypic information. With regard to T1D [68], more than 40 genomic regions have been reported to be associated with the disease, and the human HLA genes are on the top of the list with *P* values < 4E-136. For IBD [69], more than 160 disease associated loci have been identified and markers in the *IL23R* region have P values < 1E-160. For both T1D and IBD, large sample sizes with approximately 10,000 patients in each case and significant contribution from the MHC region, are responsible for these successes and consequent superior disease risk prediction. Therefore with data from more AN samples in addition to the current 3940, it is highly likely that we will see better results for both the GWA effort and machine learning based risk prediction. Research into schizophrenia genetics provides a similar example in which large datasets led to the breakthrough of 128 independent genome-wide association signals [70] following identification of marginal hits with a few thousand cases in early stages [71].

In this report, we compared multiple machine learning methods LR, SVM and GBM. Results suggested that LR and SVM are similar in terms of AUC values, while GBM showed lower performance. From a practical perspective, the LR results are promising and LR models are easier to interpret and construct than SVM, although the current result requires improvement if genotype based AN risk prediction is to be used clinically. GBT is good at capturing interacting effects and may not be optimal when true models are linear. We have many SNPs typed thus it is likely interacting SNPs, if any, may already be interrogated well by a single SNP. In addition, here the number of SNPs is much larger than the sample size; a simple linear model may be more robust to over-fitting than assuming complex tree structures. We also tried to use random forests (RF) as well, but due to high time complexity and slow convergence this methods was excluded from the analysis. Further investigation is required to assess the performance of RF. Moreover, we can compare these methods with different parameter combinations and settings when a larger cohort of AN samples are available.

We shuffled LR model 10 times and got variable number of SNPs as predictors, with the observation that three SNPs are always in the shuffles (rs9982741, rs6092077 and rs2230513), and two SNPs (rs10250561 and rs16835204) are in 9 of the 10 shuffles. Those SNPs have the highest p values (from 4.39E-6 to 3.05E-4) in the fold1 GWA study. In light of AN’s high heritability [20–26] and current lack of genome-wide significant markers [35–37], we anticipate that collating, phenotyping, genotyping and possibly sequencing more AN cases will reveal more strongly associated SNPs (thus serving as representative predictors), and greatly improve the performance of the machine learning models with higher specificity and sensitivity, which could be highly useful in a clinical setting. More complicated models including copy number variation, rare variants and even environmental factors will also lead to better performance. This and the discovery of significantly associated genomic loci or other biomarkers, will bring us closer to the goal of individualized medicine through early diagnosis and intervention for AN.

## Conclusion

Using machine learning techniques, here we present the first AN risk prediction study based on genome wide genotype data. Our results indicated higher performances of LR and SVM as opposed to GBT, with the greatest discriminative value AUC being 0.693 for the linear model. In addition, we showed that larger sample sizes can improve the machine learning risk prediction outcome, urging expanded AN case collection through international collaboration. With more genomic and other data in a greater sample pool, our study and the motheds we used will serve as the first step toward genomic screening of AN risk in a clinical setting.

### Availability of supporting data

Anorexia nervosa case summary statistics can be found at the PGC website (https://www.med.unc.edu/pgc/downloads).

## Additional files

Additional file 1 **Table S1.** Performance of logistic regression model in 10 random shuffles. **Table S2.** AUCs for fraction of the training dataset (from 10 % to 90 % of the original), after rerunning 10 times. **Table S3.** AUCs of different size for training dataset (from 10 % more to 90 % more than the original, randomly selected from fold3), after rerunning 10 times. Member lists of the Genetic Consortium for Anorexia Nervosa (GCAN)/the Wellcome Trust Case Control Consortium 3 (WTCCC 3)/the Price Foundation Collaborative Group. Supplementary acknowledgements. Ethic committee information. (DOCX 45 kb)

Additional file 2: Figure S1. ROC curve for linear regression model in shuffle 1. (PDF 17 kb)

## Abbreviations

AN: Anorexia Nervosa
AUC: Area Under the receiver operating characteristic (ROC) Curve
GBT: Gradient Boosted Trees
GWA: genome wide association
IBD: Inflammatory Bowel Disease
LR: logistic regression
SNP: Single Nucleotide Polymorphism
SVM: Support Vector Machine
T1D: Type I Diabetes
