# Supplementary material for: Machine learning derived risk prediction of anorexia nervosa
Source: BMC Med Genomics. 2016 Jan 20;9:4. doi: 10.1186/s12920-016-0165-x (PMC4721143; doi:10.1186/s12920-016-0165-x)
Supplement: Supplementary file 1 — Table S1. Performance of logistic regression model in 10 random shuffles. Table S2. AUCs for fraction of the training dataset (from 10 % to 90 % of the original), after rerunning 10 times. Table S3. AUCs of different size for training dataset (from 10 % more to 90 % more than the original, randomly selected from fold3), after rerunning 10 times. Member lists of the Genetic Consortium for Anorexia Nervosa (GCAN)/the Wellcome Trust Case Control Consortium 3 (WTCCC 3)/the Price Foundation Collaborative Group. Supplementary acknowledgements. Ethic committee information. (DOCX 45 kb) [file 12920_2016_165_MOESM1_ESM.docx]

# Additional file 1

Machine Learning Derived Disease Risk Prediction for Anorexia Nervosa

Yiran Guo, Zhi Wei, Brendan Keating, The Genetic Consortium for Anorexia Nervosa, The Wellcome Trust Case Control Consortium 3, Price Foundation Collaborative Group, Hakon Hakonarson

|  | | **Table S1. Performance of logistic regression model in 10 random shuffles.** | | | | | | | | |
| --- | --- | --- | --- | --- | --- | --- | --- | --- | --- | --- |
| **Shuffle** | **Fold1 sample size*** | | **Fold2 sample size*** | **Fold3 sample size*** | **# SNPs with *P*-value < 1E-3 in fold1** | **# SNPs in LR prediction model** | **LR sensitivity/ specificity** | **LR AUC in fold3** | **SVM AUC in fold3** | **GBT AUC in fold3** |
| 1 | 1,289/3,113 | | 1,341/3,061 | 1,310/3,092 | 1,486 | 273 | 0.11/0.97 | 0.693 | 0.691 | 0.623 |
| 2 | 1,322/3,080 | | 1,299/3,103 | 1,319/3,083 | 1,429 | 264 | 0.09/0.96 | 0.677 | 0.689 | 0.577 |
| 3 | 1,359/3,043 | | 1,319/3,083 | 1,262/3,140 | 1,281 | 219 | 0.07/0.99 | 0.706 | 0.723 | 0.604 |
| 4 | 1,290/3,112 | | 1,283/3,119 | 1,367/3,035 | 1,407 | 240 | 0.04/0.99 | 0.675 | 0.687 | 0.592 |
| 5 | 1,295/3,107 | | 1,314/3,088 | 1,331/3,071 | 1,312 | 287 | 0.13/0.96 | 0.691 | 0.685 | 0.613 |
| 6 | 1,290/3,112 | | 1,315/3,087 | 1,335/3,067 | 1,268 | 184 | 0.02/0.99 | 0.696 | 0.706 | 0.603 |
| 7 | 1,278/3,124 | | 1,312/3,090 | 1,350/3,052 | 1,121 | 214 | 0.05/0.99 | 0.683 | 0.680 | 0.606 |
| 8 | 1,304/3,098 | | 1,302/3,100 | 1,334/3,068 | 1,309 | 137 | 0.00/1.00 | 0.681 | 0.695 | 0.581 |
| 9 | 1,314/3,088 | | 1,301/3,101 | 1,325/3,077 | 1,373 | 154 | 0.00/1.00 | 0.669 | 0.702 | 0.579 |
| 10 | 1,328/3,074 | | 1,300/3,102 | 1,312/3,090 | 1,568 | 208 | 0.02/0.99 | 0.684 | 0.690 | 0.568 |
|  |  | |  |  |  |  |  | df | 18 | 15 |
|  |  | |  |  |  |  |  | t stat | -1.76 | 13.66 |
|  |  | |  |  |  |  |  | *P*-value | 0.096 | **7.2E-10** |
|  | | LR: Logistic Regression. SVM: Support Vector Machine. GBT: Gradient Boosted Trees. AUC: Area Under the (receiver operating characteristic) Curve. *Number of cases / number of controls. | | | | | | | | |

| **Table S2. AUCs for fraction of the training dataset (from 10% to 90% of the original), after rerunning 10 times. Significant *P*-values are in bold.** | | | | | | | | | |
| --- | --- | --- | --- | --- | --- | --- | --- | --- | --- |
| **Fraction of fold2** | **10%** | **20%** | **30%** | **40%** | **50%** | **60%** | **70%** | **80%** | **90%** |
| Rerun_1 | 0.526 | 0.584 | 0.625 | 0.626 | 0.636 | 0.662 | 0.681 | 0.680 | 0.684 |
| Rerun_2 | 0.547 | 0.600 | 0.600 | 0.645 | 0.635 | 0.659 | 0.656 | 0.672 | 0.688 |
| Rerun_3 | 0.536 | 0.599 | 0.634 | 0.638 | 0.660 | 0.654 | 0.674 | 0.680 | 0.681 |
| Rerun_4 | 0.539 | 0.582 | 0.611 | 0.594 | 0.671 | 0.667 | 0.679 | 0.680 | 0.686 |
| Rerun_5 | 0.535 | 0.584 | 0.632 | 0.622 | 0.652 | 0.649 | 0.674 | 0.677 | 0.694 |
| Rerun_6 | 0.553 | 0.613 | 0.606 | 0.628 | 0.623 | 0.668 | 0.670 | 0.682 | 0.688 |
| Rerun_7 | 0.572 | 0.612 | 0.590 | 0.634 | 0.672 | 0.647 | 0.680 | 0.687 | 0.689 |
| Rerun_8 | 0.574 | 0.576 | 0.588 | 0.603 | 0.664 | 0.651 | 0.664 | 0.672 | 0.688 |
| Rerun_9 | 0.558 | 0.598 | 0.635 | 0.608 | 0.661 | 0.665 | 0.674 | 0.692 | 0.691 |
| Rerun_10 | 0.522 | 0.558 | 0.606 | 0.644 | 0.650 | 0.655 | 0.677 | 0.672 | 0.692 |
| df | 9 | 9 | 9 | 9 | 9 | 9 | 9 | 9 | 9 |
| t stat | -25.86 | -19.01 | -14.26 | -12.43 | -7.80 | -14.66 | -8.15 | -6.50 | -4.06 |
| *P*-values | **9.31E-10** | **1.41E-08** | **1.75E-07** | **5.70E-07** | **2.71E-05** | **1.38E-07** | **1.90E-05** | **1.12E-04** | **2.83E-03** |

| **Table S3. AUCs of different size for training dataset (from 10% more to 90% more than the original, randomly selected from fold3), after rerunning 10 times. Significant *P-*values are in bold.** | | | | | | | | | |
| --- | --- | --- | --- | --- | --- | --- | --- | --- | --- |
| **Multiple of fold2** | **1.1X** | **1.2X** | **1.3X** | **1.4X** | **1.5X** | **1.6X** | **1.7X** | **1.8X** | **1.9X** |
| Rerun_1 | 0.697 | 0.693 | 0.691 | 0.699 | 0.694 | 0.716 | 0.698 | 0.702 | 0.659 |
| Rerun_2 | 0.700 | 0.692 | 0.691 | 0.690 | 0.690 | 0.701 | 0.691 | 0.699 | 0.698 |
| Rerun_3 | 0.690 | 0.699 | 0.691 | 0.697 | 0.699 | 0.703 | 0.699 | 0.671 | 0.710 |
| Rerun_4 | 0.698 | 0.691 | 0.687 | 0.689 | 0.685 | 0.697 | 0.690 | 0.711 | 0.732 |
| Rerun_5 | 0.694 | 0.702 | 0.689 | 0.706 | 0.704 | 0.711 | 0.707 | 0.697 | 0.692 |
| Rerun_6 | 0.693 | 0.687 | 0.691 | 0.701 | 0.693 | 0.712 | 0.687 | 0.707 | 0.733 |
| Rerun_7 | 0.690 | 0.696 | 0.702 | 0.690 | 0.690 | 0.694 | 0.711 | 0.745 | 0.714 |
| Rerun_8 | 0.698 | 0.697 | 0.694 | 0.707 | 0.707 | 0.694 | 0.703 | 0.721 | 0.733 |
| Rerun_9 | 0.701 | 0.705 | 0.695 | 0.697 | 0.688 | 0.698 | 0.723 | 0.714 | 0.745 |
| Rerun_10 | 0.694 | 0.696 | 0.695 | 0.695 | 0.703 | 0.689 | 0.721 | 0.727 | 0.709 |
| df | 9 | 9 | 9 | 9 | 9 | 9 | 9 | 9 | 9 |
| t stat | -2.03 | -1.66 | 0.30 | -2.03 | -0.97 | -3.02 | -2.52 | -2.62 | -2.43 |
| *P*-values | 0.073 | 0.132 | 0.768 | 0.073 | 0.358 | **0.015** | **0.033** | **0.028** | **0.038** |

**Figure Legends**

**Figure S1. ROC curve for linear regression model in shuffle 1.**

# Membership of The Genetic Consortium for Anorexia Nervosa (GCAN)

V Boraska^1,2^, CS Franklin^1^, JAB Floyd^1,3^, LM Thornton^4^, LM Huckins^1^, L Southam, BSc^1^, N William Rayner^1,5,6^, I Tachmazidou^1^, KL Klump^7^, J Treasure^8^, CM Lewis^9^, U Schmidt^8^, F Tozzi^4^, K Kiezebrink^10^, J Hebebrand^11^, P Gorwood^12,13^, RAH Adan^14,15^, MJH Kas^14^, AFavaro^16^, P Santonastaso^16^, F Fernández-Aranda^17,18^, M Gratacos^19,20,21,22^, F Rybakowski^23^, M Dmitrzak-Weglarz^24^, J Kaprio^25,26,27^, A Keski-Rahkonen, MPH^25^, A Raevuori^25,28^, EF Van Furth^29,30^, MCT Slof-Op t Landt^29,31^, JI Hudson^32^, T Reichborn-Kjennerud^33,34^, GPS Knudsen^33^, P Monteleone^35,36^, AS Kaplan^37,38^, A Karwautz^39^, H Hakonarson^40,41^, WH Berrettini^42^, Y Guo^40^, D Li^40^, NJ Schork^43^, G Komaki^44,45^, T Ando^44^, H Inoko^46^, T Esko^47^, K Fischer^47^, K Männik^48,49^, A Metspalu^47,48^, JH Baker^4^, RD Cone^50^, J Dackor^51^, JE DeSocio^52^, CE Hilliard^4^, JK O'Toole^53^, J Pantel^54^, JP Szatkiewicz^51^, C Taico^4^, S Zerwas^4^, SE Trace^4^, OSP Davis^9,55^, S Helder^9^, K Bühren^56^, R Burghardt^57^, M de Zwaan^58,59^, K Egberts^60^, S Ehrlich^61,62^, B Herpertz-Dahlmann^56^, W Herzog^63^, H Imgart^64^, A Scherag^65^, S Scherag^11^, S Zipfel^66^, C Boni^12^, N Ramoz^12^, A Versini^12^, MK Brandys^14,15^, UN Danner^15^, C de Kovel^67^, J Hendriks^14^, BPC Koeleman^67^, RA Ophoff^68,69^, E Strengman^67^, AA van Elburg^15,70^, A Bruson^71^, M Clementi^71^, D Degortes^16^, M Forzan^71^, E Tenconi^16^, E Docampo^19,20,21,22^, G Escaramís^19,20,21,22^, S Jiménez-Murcia^17,18^, J Lissowska^72^, A Rajewski^73^, N Szeszenia-Dabrowska^73^, A Slopien^24^, J Hauser^24^, L Karhunen^74^, I Meulenbelt^31^, PE Slagboom^31,75^, A Tortorella^35^, M Maj^35^, G Dedoussis^76^, D Dikeos^77^, F Gonidakis^78^, K Tziouvas^76^, A Tsitsika^79^, H Papezova^80^, L Slachtova^81^, D Martaskova^80^, JL Kennedy^37,38^, RD Levitan^37,38^, Z Yilmaz^4,37^, J Huemer^39^, D Koubek^39^, E Merl^39^, G Wagner^39^, P Lichtenstein^82^, G Breen^9^, S Cohen-Woods^9^, A Farmer^9^, P McGuffin^9^, S Cichon^83,84,85^, I Giegling^86^, S Herms^83,85^, D Rujescu^86^, S Schreiber^87^, H-E Wichmann^88,89^, C Dina^90^, R Sladek^91^, G Gambaro^92^, N Soranzo^1^, A Julia^93^, S Marsal^93^, Ra Rabionet^19,20,21,22^, V Gaborieau^94^, DM Dick^95^, A Palotie^1,96,97^, S Ripatti^96,98^, E Widén^96,98^, OA Andreassen^99^, T Espeseth^99,100^, A Lundervold^101,102,103^, I Reinvang^100^, VM Steen^104,105^, S Le Hellard^104,105^, M Mattingsdal^99^, I Ntalla^76^, V Bencko^106^, L Foretova^107^, V Janout^108^, M Navratilova^107^, S Gallinger^109^, D Pinto^110^, SW Scherer^111^, H Aschauer^112^, L Carlberg^112^, A Schosser^112^, L Alfredsson^113^, B Ding^113^, L Klareskog^114^, L Padyukov^114^, C Finan^1^, G Kalsi^9^, M Roberts^9^, DW Logan^1^, L Peltonen^1^, GRS Ritchie^1,115^, P Courtet^116,117^, S Guillame^116,117^, I Jaussent^116,117^, JC Barrett^1^, X Estivill^19,20,21,22^, A Hinney^11^, PF Sullivan, FRANZCP^4,51^, DA Collier^9,118^, E Zeggini^1^, CM Bulik^4,119^

**Affiliations**

1 Wellcome Trust Sanger Institute, Wellcome Trust Genome Campus, Hinxton, Cambridge, UK

2 University of Split School of Medicine, Split, Croatia

3 William Harvey Research Institute, Barts and The London School of Medicine and Dentistry, Queen Mary University of London, John Vane Science Centre, Charterhouse Square, London, UK

4 Department of Psychiatry, University of North Carolina at Chapel Hill, Chapel Hill, NC, USA

5 Wellcome Trust Centre for Human Genetics (WTCHG), University of Oxford, Oxford, UK

6 Oxford Centre for Diabetes, Endocrinology and Metabolism (OCDEM), Oxford, UK

7 Department of Psychology, Michigan State University, East Lansing, MI, USA

8 Section of Eating Disorders, Institute of Psychiatry, King’s College London, London, UK

9 Social, Genetic and Developmental Psychiatry Centre, Institute of Psychiatry, King’s College London, London, UK

10 Health Services Research Unit, University of Aberdeen, Aberdeen, UK

11 Department of Child and Adolescent Psychiatry, Psychosomatics and Psychotherapy, Universitätsklinikum Essen, University of Duisburg-Essen, Essen, Germany

12 INSERM U894, Centre of Psychiatry and Neuroscience, Paris, France

13 Sainte-Anne Hospital (CMME), University of Paris-Descartes, Paris, France

14 Brain Center Rudolf Magnus, Department of Translational Neuroscience, University Medical Center Utrecht, Utrecht, The Netherlands

15 Altrecht Eating Disorders Rintveld, Zeist, The Netherlands

16 Department of Neurosciences, University of Padova, Padova, Italy

17 Department of Psychiatry and CIBERON, University Hospital of Bellvitge-IDIBELL, Barcelona, Spain

18 Department of Clinical Sciences, School of Medicine, University of Barcelona, Barcelona, Spain

19 Genomics and Disease Group, Centre for Genomic Regulation (CRG), Barcelona, Spain

20 Universitat Pompeu Fabra (UPF), Barcelona, Spain

21 Centro de Investigación Biomédica en Red en Epidemiología y Salud Pública (CIBERESP), Barcelona, Spain

22 Hospital del Mar Medical Research Institute (IMIM), Barcelona, Spain

23 Department of Child and Adolescent Psychiatry, Institute of Psychiatry and Neurology, Warsaw, Poland

24 Department of Child and Adolescent Psychiatry, Department of Psychiatry, Poznan University of Medical Sciences, Poznan, Poland

25 Hjelt Institute, University of Helsinki, Helsinki, Finland

26 Institute of Molecular Medicine, University of Helsinki, Helsinki, Finland

27 Department of Mental Health and Substance Abuse Services, National Institute for Health and Welfare, Helsinki, Finland

28 Department of Adolescent Psychiatry, Helsinki University Central Hospital, Helsinki, Finland

29 Center for Eating Disorders Ursula, Leiden, The Netherlands

30 Leiden University Medical Centre, Department of Psychiatry, Leiden, The Netherlands

31 Leiden University Medical Centre, Molecular Epidemiology Section (Department of Medical Statistics), Leiden, The Netherlands

32 Department of Psychiatry, McLean Hospital/Harvard Medical School, Belmont, MA, USA

33 Department of Genetics, Environment and Mental Health, Norwegian Institute of Public Health, Oslo, Norway

34 Institute of Clinical Medicine, University of Oslo, Oslo, Norway

35 Department of Psychiatry, University of Naples SUN, Naples, Italy

36 Chair of Psychiatry, University of Salerno, Salerno, Italy

37 Centre for Addiction and Mental Health, University of Toronto, Toronto, Canada

38 Department of Psychiatry, University of Toronto, Toronto, Canada

39 Eating Disorders Unit, Department of Child and Adolescent Psychiatry, Medical University of Vienna, Vienna, Austria

40 The Center for Applied Genomics, The Children's Hospital of Philadelphia, Philadelphia, PA, USA

41 The Division of Human Genetics, Department of Pediatrics, The Perelman School of Medicine, University of Pennsylvania, Philadelphia, PA, USA

42 Department of Psychiatry, University of Pennsylvania, Philadelphia, PA, USA

43 Department of Molecular and Experimental Medicine and The Scripps Translational Science Institute, The Scripps Research Institute, La Jolla, CA, USA

44 Department of Psychosomatic Research, National Institute of Mental Health, NCNP, Tokyo, Japan

45 School of Health Sciences at Fukuoka, International University of Health and Welfare, Fukuoka, Japan

46 Department of Molecular Life Sciences, Tokai University School of Medicine, Kanagawa, Japan

47 Estonian Genome Center, University of Tartu, Tartu, Estonia

48 Institute of Molecular and Cell Biology, University of Tartu, Tartu, Estonia

49 Center for Integrative Genomics, University of Lausanne, Lausanne, Switzerland

50 Department of Molecular Physiology and Biophysics, Vanderbilt University School of Medicine, Nashville, TN, USA

51 Department of Genetics, The University of North Carolina at Chapel Hill, Chapel Hill, NC, USA

52 Seattle University College of Nursing, Seattle, WA, USA

53 Kartini Clinic, Portland, OR, USA

54 Centre de Psychiatrie et Neurosciences – Inserm U894, Paris, France

55 UCL Genetics Institute, Department of Genetics, Evolution and Environment, University College London, London, UK

56 Department of Child and Adolescent Psychiatry, Psychosomatics and Psychotherapy, University Clinics RWTH Aachen, Aachen, Germany

57 Department of Child and Adolescent Psychiatry, Psychosomatics and Psychotherapy, Charité, Berlin, Germany

58 Department of Psychosomatic Medicine and Psychotherapy, Hannover Medical School, Hannover, Germany

59 Department of Psychosomatic Medicine and Psychotherapy, University of Erlangen-Nuremberg, Erlangen, Germany

60 Department of Child and Adolescent Psychiatry, Psychosomatics and Psychotherapy, University Würzburg, Würzburg, Germany

61 Department of Child and Adolescent Psychiatry, University Hospital Carl Gustav Carus, Dresden University of Technology, Dresden, Germany

62 Massachusetts General Hospital/Harvard Medical School, Athinoula A. Martinos Center for Biomedical Imaging, Psychiatric Neuroimaging Research Program, Charlestown, MA, USA

63 Departments of Psychosocial and Internal Medicine, Heidelberg University, Heidelberg, Germany

64 Parklandklinik, Bad Wildungen, Germany

65 Institute for Medical Informatics, Biometry and Epidemiology, Universitätsklinikum Essen, University of Duisburg-Essen, Essen, Germany

66 Department of Internal Medicine VI, Psychosomatic Medicine and Psychotherapy, University Medical Hospital Tübingen, Tübingen, Germany

67 Department of Medical Genetics, University Medical Center Utrecht, Utrecht, The Netherlands

68 Center for Neurobehavioral Genetics, University of California, Los Angeles, Los Angeles, CA, USA

69 Brain Center Rudolf Magnus, Department of Psychiatry, University Medical Center Utrecht, The Netherlands

70 Department of Social Sciences, Utrecht University, Utrecht, The Netherlands

71 Clinical Genetics Unit, Department of Woman and Child Health, University of Padova, Padova, Italy

72 M. Sklodowska-Curie Cancer Center and Institute of Oncology, Warsaw, Poland

73 Department of Epidemiology, Institute of Occupational Medicine, Department of Epidemiology, Lodz, Poland

74 Department of Clinical Nutrition, Institute of Public Health and Clinical Nutrition, University of Eastern Finland, Kuopio, Finland

75 Netherlands Consortium for Healthy Ageing, Leiden University Medical Center, The Netherlands

76 Department of Nutrition and Dietetics, Harokopio University, Athens, Greece

77 1st Department of Psychiatry, Athens University Medical School, Athens, Greece

78 Eating Disorders Unit, 1st Department of Psychiatry, Athens University Medical School, Athens, Greece

79 Adolescent Health Unit (AHU), 2nd Department of Pediatrics – Medical School, University of Athens ‘P & A Kyriakou’ Children's Hospital, Athens, Greece

80 Department of Psychiatry, 1st Faculty of Medicine, Charles University, Prague, Czech Republic

81 Department of Pediatrics, 1st Faculty of Medicine, Charles University, Prague, Czech Republic

82 Department of Medical Epidemiology and Biostatistics, Karolinska Institutet, Stockholm, Sweden

83 Institute of Human Genetics, Department of Genomics, Life & Brain Center, University of Bonn, Bonn, Germany

84 Institute of Neuroscience and Medicine (INM-1), Research Center Jülich, Jülich, Germany

85 Division of Medical Genetics, Department of Biomedicine, University of Basel, Basel, Switzerland

86 Martin-Luther-Universität Halle-Wittenberg, Klinikum der Medizinischen Fakultät, Halle/Saale, Germany

87 Institute of Clinical Molecular Biology, University of Kiel, Kiel, Germany

88 Institute of Epidemiology, Helmholtz Zentrum München, German Research Center for Environmental Health, Neuherberg, Germany

89 Institute of Medical Informatics, Biometry and Epidemiology, Ludwig-Maximilians-University, Munich, Germany

90 CNRS 8090-Institute of Biology, Pasteur Institute, Lille, France

91 McGill University and Genome Quebec Innovation Centre, Montreal, QC, Canada

92 Division of Nephrology, Department of Internal Medicine and Medical Specialties, Columbus-Gemelly Hospitals, Catholic University, Rome, Italy

93 Unitat de Recerca de Reumatologia (URR), Institut de Recerca Hospital Universitari Vall d'Hebron, Barcelona, Spain

94 Genetic Epidemiology Group, International Agency for Research on Cancer (IARC), Lyon, France

95 Virginia Institute for Psychiatric and Behavioral Genetics, Department of Psychiatry, Virginia Commonwealth University, Virginia, VA, USA

96 The Finnish Institute of Molecular Medicine Finland (FIMM), University of Helsinki, Helsinki, Finland

97 The Program for Human and Population Genetics, The Broad Institute of MIT and Harvard, Cambridge, MA, USA

98 Finnish Institute of Occupational Health, Province of Southern Finland, Helsinki, Finland

99 NORMENT, KG Jebsen Centre for Psychosis Research, Division of Mental Health and Addiction, Oslo University Hospital & Institute of Clinical Medicine, University of Oslo, Oslo, Norway

100 Department of Psychology, University of Oslo, Oslo, Norway

101 Department of Biological and Medical Psychology, University of Bergen, Bergen, Norway

102 Kavli Research Centre for Aging and Dementia, Haraldsplass Deaconess Hospital, Bergen, Norway

103 K.G. Jebsen Centre for Research on Neuropsychiatric Disorders, University of Bergen, Bergen, Norway

104 KG Jebsen Centre for Psychosis Research, Norwegian Centre For Mental Disorders Research (NORMENT), Department of Clinical Science, University of Bergen, Bergen, Norway

105 Dr Einar Martens Research Group for Biological Psychiatry, Center for Medical Genetics and Molecular Medicine, Haukeland University Hospital, Bergen, Norway

106 Institute of Hygiene and Epidemiology, 1st Faculty of Medicine, Charles University, Prague, Czech Republic

107 Department of Cancer Epidemiology and Genetics, Masaryk Memorial Cancer Institute, Brno, Czech Republic

108 Palacky University, Olomouc, Czech Republic

109 University Health Network and Mount Sinai Hospital, Toronto General Hospital, and Samuel Lunenfeld Research Institute, Toronto, ON, Canada

110 Departments of Psychiatry, and Genetics and Genomic Sciences, Seaver Autism Center, and the Mindich Child Health and Development Institute, Mount Sinai School of Medicine, New York, NY, USA

111 The Centre for Applied Genomics and Program in Genetics and Genome Biology, The Hospital for Sick Children, Toronto, ON, Canada

112 Department of Psychiatry and Psychotherapy, Medical University Vienna, Vienna, Austria

113 The Institute of Environmental Medicine, Karolinska Institutet, Stockholm, Sweden

114 Rheumatology Unit, Department of Medicine at the Karolinska University Hospital, Solna, Sweden

115 European Molecular Biology Laboratory, European Bioinformatics Institute, Wellcome Trust Genome Campus, Hinxton, Cambridge, UK

116 Inserm, U1061, Université Montpellier 1, Montpellier, France

117 Department of Emergency Psychiatry, CHU Montpellier, Montpellier, France

118 Eli Lilly and Company Ltd, Erl Wood Manor, Windlesham, Surrey, UK

119 Department of Nutrition, The University of North Carolina at Chapel Hill, Chapel Hill, NC, USA

# Wellcome Trust Sanger Institute The Wellcome Trust Case Control Consortium 3 (WTCCC 3)

*Data Analysis Group*: Carl A Anderson^1^, Jeffrey C Barrett^1^, James AB Floyd^1^, Christopher S Franklin^1^, Ralph McGinnis^1^, Nicole Soranzo^1^, Eleftheria Zeggini^1^.

*UK Blood Services Controls*: Jennifer Sambrook^2^, Jonathan Stephens^2^, Willem H Ouwehand^2^.

*1958 Birth Cohort Controls*: Wendy L McArdle^3^, Susan M Ring^3^, David P Strachan^4^.

*Management Committee*: Graeme Alexander^5^, Cynthia M Bulik^6^, David A Collier^7^, Peter J Conlon^8^, Anna Dominiczak^9^, Audrey Duncanson^10^, Adrian Hill^11^, Cordelia Langford^1^, Graham Lord^12^, Alexander P Maxwell^13^, Linda Morgan^14^, Leena Peltonen^1^, Richard N Sandford^15^, Neil Sheerin^12^, Nicole Soranzo^1^, Fredrik O Vannberg^11^, Jeffrey C Barrett^1^ (chair).

*DNA, Genotyping, and Informatics Group*: Hannah Blackburn^1^, Wei-Min Chen^16^, Sarah Edkins^1^, Mathew Gillman^1^, Emma Gray^1^, Sarah E Hunt^1^, Cordelia Langford^1^, Suna Onengut-Gumuscu^16^, Simon Potter^1^, Stephen S Rich^16^, Douglas Simpkin^1^, Pamela Whittaker^1^.

**Affiliations**

1. The Wellcome Trust Sanger Institute, Hinxton, Cambridge CB10 1SA, UK

2. Division of Transfusion Medicine, Department of Haematology, University of Cambridge, NHSBT Cambridge Centre, Long Road, Cambridge CB2 0PT, UK

3. Department of Social Medicine, University of Bristol, Bristol BS8 2BN, UK

4. St George’s University, Division of Community Health Sciences, London SW19 0RE, UK

5. Department of Hepatology, Cambridge University Hospitals NHS Foundation Trust, Cambridge CB2 0QQ, UK

6. Department of Psychiatry, University of North Carolina at Chapel Hill, Chapel Hill, NC, USA

7. Institute of Psychiatry, King’s College London, London SE5 8AF, UK

8. Department of Nephrology, Beaumont Hospital, Dublin, Ireland and Royal College of Surgeons Dublin, Dublin, Ireland

9. BHF Glasgow Cardiovascular Research Centre, University of Glasgow, Glasgow G12 8TA, UK

10. Gibbs Building, 215 Euston Road, London NW1 2BE, UK

11. Wellcome Trust Centre for Human Genetics, University of Oxford, Oxford OX1 2JA, UK

12. MRC Centre for Transplantation, King’s College London, London SE1 9RT, UK

13. Belfast City Hospital, Lisburn Road, Belfast BT9 7AB, UK

14. School of Molecular Medical Sciences, University of Nottingham, Nottingham NG7 2UH, UK

15. Academic Department of Medical Genetics, Cambridge University, Cambridge CB2 0QQ, UK

16. Center for Public Health Genomics, University of Virginia, Charlottesville, VA, USA.

# The Price Foundation Collaborative Group

Harry Brandt^1^, Steve Crawford^1^, Scott Crow^2^, Manfred M. Fichter^3,4^, Katherine A. Halmi^5^, Craig Johnson^6^, Allan S. Kaplan^7,8^, Maria La Via^9^, James Mitchell^10,11^, Michael Strober^12^, Alessandro Rotondo^13^, Janet Treasure^14^, D. Blake Woodside^7,8,15^, Cynthia M. Bulik^16^, Pamela Keel^17^, Kelly L. Klump^18^, Lisa Lilenfeld^19^, Laura M. Thornton^16^, Kathy Plotnicov^20^, Andrew W. Bergen^21^, Wade Berrettini^22^, Walter Kaye^23^, and Pierre Magistretti^24^.

**Affiliations**

1. Department of Psychiatry, University of Maryland School of Medicine, Baltimore, MD, USA.

2. Department of Psychiatry, University of Minnesota, Minneapolis, MN, USA.

3. Roseneck Hospital for Behavioral Medicine, Prien, Germany.

4. Department of Psychiatry, University of Munich (LMU), Munich, Germany.

5. New York Presbyterian Hospital, Westchester Division, Weill Medical College of Cornell University, White Plains, NY, USA.

6. Laureate Psychiatric Clinic and Hospital, Tulsa, OK, USA.

7. Center for Addiction and Mental Health, Toronto, Canada.

8. Department of Psychiatry, Toronto General Hospital, University Health Network, Toronto, Canada.

9. Department of Psychiatry, University of North Carolina at Chapel Hill, Chapel Hill, NC, USA.

10. Neuropsychiatric Research Institute, Fargo, ND, USA.

11. Department of Clinical Neuroscience, University of North Dakota School of Medicine and Health Sciences, Grand Forks, ND, USA.

12. Department of Psychiatry and Biobehavioral Sciences, David Geffen School of Medicine, University of California at Los Angeles, Los Angeles, CA, USA.

13. Neuropsychiatric Research Biotechnologies, University of Pisa, Pisa, Italy.

14. Eating Disorders Section, Institute of Psychiatry, King's College, University of London, London, England.

15. Department of Psychiatry, University of Toronto, Toronto, Canada.

16. Department of Psychiatry, University of North Carolina at Chapel Hill, Chapel Hill, NC, USA.

17. Department of Psychology, Florida State University, Tallahassee, FL, USA.

18. Department of Psychology, Michigan State University, East Lansing, MI, USA.

19. Department of Psychology, Georgia State University, Atlanta, GA, USA.

20. Eating Disorders Module, Western Psychiatric Institute & Clinic, University of Pittsburgh School of Medicine, Pittsburgh, PA, USA.

21. Center for Health Sciences, SRI International, Menlo Park, CA, USA.

22. Department of Psychiatry, University of Pennsylvania, Philadelphia, PA, USA.

23. Department of Psychiatry, University of California at San Diego, San Diego, CA, USA.

24. Department of Psychiatry, Brain Mind Institute EPFL—Lausanne, Center for Psychiatric Neuroscience, University of Lausanne Medical School, Lausanne, Switzerland.

# Supplementary acknowledgements

**Acknowledgment for funding, biomaterials, and clinical data**

This work was funded by a grant from the WTCCC3 WT088827/Z/09 entitled ‘A genome-wide association study of anorexia nervosa’.

**Wellcome Trust acknowledgments**

This work was supported by the WellcomeTrust (098051). Eleftheria Zeggini is supported by the Wellcome Trust (098051). Vesna Boraska is supported by Unity Through Knowledge Fund CONNECTIVITY PROGRAM (‘Gaining Experience’ Grant 2A), The National Foundation for Science, Higher Education and Technological Development of the Republic of Croatia (BRAIN GAIN- Postdoc fellowship) and the Wellcome Trust (098051). Christopher S Franklin is supported by the WTCCC3 project, which is supported by the Wellcome Trust (WT090355/A/09/Z, WT090355/B/09/Z). James A B Floyd is supported by the WTCCC3 project, which is supported by the Wellcome Trust (WT090355/A/09/Z, WT090355/B/09/Z). Lorraine Southam is supported by the Wellcome Trust (098051). William N Rayner is supported by the Wellcome Trust (098051). The Wellcome Trust Case Control Consortium 3 project is supported by the Wellcome Trust (WT090355/A/09/Z, WT090355/B/09/Z). We acknowledge use of data from the British 1958 Birth Cohort and the UK National Blood Service. We obtained High Density SNP Association Analysis of Melanoma: Case-Control and Outcomes Investigation data set through dbGaP (dbGaP Study Accession: phs000187.v1.p1). Research support to collect data and develop an application to support this project was provided by 3P50CA093459, 5P50CA097007, 5R01ES011740 and 5R01CA133996. Laura Huckins acknowledges Wellcome Trust (098051) and the MRC (MR/J500355/1) and Ximena Ibarra-Soria for advice on RNA-seq analysis.

**Acknowledgments for anorexia nervosa cases**

Austria, Medical University of Vienna: The study was partly supported by the European Commission, Framework 5 research program, Integrated Project QLK1-CT-1999-00916 ‘Factors in Healthy Eating’ given to the consortium lead by Professor J Treasure and Professor D Collier, London. We thank Gerald Nobis, Dr Maria Haidvogl and Dr Julia Philipp for help with data collection and interview work.

Canada, Canadian Institutes of Health Research (CIHR): Zeynep Yilmaz was supported by a CIHR Doctoral Research Award (Genetic Determinants of Low Body Weight in Anorexia Nervosa; funding reference: GSD-111968). The Toronto authors thank Sajid Shaikh, Maria Tampakeras and Natalie Freeman for DNA preparation and laboratory support.

Canada, The Ontario Mental Health Foundation (OMHF): The collection of the Toronto DNA samples was supported by a grant from the OMHF, awarded to Allan S Kaplan and Robert D Levitan (Polymorphism in Serotonin System Genes: Putative Role in Increased Eating Behaviour in Seasonal Affective Disorder and Bulimia Nervosa).

Czech Republic, Charles University: The study was supported by Grants IGA MZ ČR NS/10045-4 and IGA NT 14094/3 from the Czech Ministry of Education and Health and PRVOUK P24/LF1/3 and P26/LF1/4 Charles University, Prague, and from the Marie Curie Research Training Network INTACT (MRTN-CT-2006-035988).

Finland, University of Helsinki: Academy of Finland Center of Excellence in Complex Disease Genetics (Grant numbers: 213506, 129680), ENGAGE—European Network for Genetic and Genomic Epidemiology, FP7-HEALTH-F4-2007, Grant agreement number 201413. Data collection in the Finnish Twin studies has been supported by the National Institute of Alcohol Abuse and Alcoholism (Grants AA-12502, AA-00145 and AA-09203 to RJ Rose and AA15416 and K02AA018755 to DM Dick), the Academy of Finland (Grants 100499, 205585, 118555 and 141054, 265240 and 264146 to JK). AR and LK were supported by the Academy of Finland, Grants 259764 and 28327, respectively.

France, Institut National de la Santé et de la Recherche Médicale (INSERM), France: This French cohort was recruited with grants from EC Framework V ‘Factors in Healthy Eating’ (a consortium coordinated by Janet Treasure and David Collier, King’s College London), and from INRA/INSERM (4M406D), and the participation of Audrey Versini’s work was supported by grants from ‘Région Ile-de-France’. Cases were ascertained from Sainte-Anne Hospital (Paris) and Robert Debre Hospital (Paris).

Genetics of Anorexia Nervosa (GAN), National Institute of Mental Health: The data and collection of biomaterials for the GAN study have been supported by the National Institutes of Health Grants (MH066122, MH066117, MH066145, MH066296, MH066147, MH0662, MH066193, MH066287, MH066288 and MH066146). The principal investigators and co-investigators of this study were University of Pittsburgh, Pittsburgh, PA: Walter Kaye, M.D., Bernie Devlin, Ph.D.; University of North Carolina at Chapel Hill, Chapel Hill, NC: Cynthia M Bulik, Ph.D.; Roseneck Hospital for Behavioral Medicine, Prien and Department of Psychiatry, University of Munich, Germany: Manfred M Fichter, M.D.; Kings College London, Institute of Psychiatry, London, UK: Janet Treasure, M.D.; Toronto General Hospital, Toronto, Ontario, Canada: Allan Kaplan, M.D., D. Blake Woodside, M.D.; Laureate Psychiatric Hospital, Tulsa, OK: Craig L. Johnson, Ph. D.; Weill Cornell Medical College, White Plains, NY: Katherine Halmi, M.D.; Sheppard Pratt Health System, Towson: Harry A. Brandt, M.D., Steve Crawford, M.D.; Neuropsychiatric Research Institute, Fargo, ND; James E. Mitchell, M.D.; University of California at Los Angeles, Los Angeles, CA: Michael Strober, Ph.D.; University of Pennsylvania, Philadelphia, PA: Wade Berrettini, M.D., Ph.D.; and University of Birmingham, England: Ian Jones, M.D. We are indebted to the participating families for their contribution of time and effort in support of this study. We thank the Price Foundation for sponsoring the earlier work of this collaboration and also thank the study managers and clinical interviewers for their efforts in participant screening and clinical assessments.

Germany, University of Duisburg-Essen: Sample collection was funded by grants from the German Federal Ministry of Education and Research (BMBF; EDNET 01GV0602, 01GV0624, 01GV0623 and 01GV0905, NGFNplus: 01GS0820) and the IFORES program of the University of Duisburg-Essen. The funders had no role in study design, data collection and analysis, decision to publish or preparation of the manuscript.

Germany, Professor Ehrlich’s work is supported by DFG Grant EH 367/5-1 and the SFB 940.

GlaxoSmithKline (GSK), Leeds (Yorkshire Centre for Eating Disorders): We acknowledge the support of the Medical Research Council and GlaxoSmithKline for providing financial support of this project. The support of the Carnegie Trust in the form of a travel award is also acknowledged. We also acknowledge the help and support of the Discovery and Pipeline Genetics, and Translational Medicine and Genetics departments at GSK for their contributions to this study. In particular, they also acknowledge Mike Stubbins, Julia Perry, Sarah Bujac, David Campbell (at GSK currently or at the time when the study was performed), John Blundell (Leeds University) and Evleen Mann (Yorkshire Centre for Eating Disorders), for their fundamental contribution to the realization of this study.

Greece, Eating Disorders Unit, 1st Department of Psychiatry, Athens University, Medical School. Special thanks goes to Associate Professor Varsou E, Head of Eating Disorders Unit, and Professor Papadimitriou G, Chairman and Director of 1st Department of Psychiatry, Athens University, Medical School, for their advice and support.

Italy, Padua (BIOVEDA): BIOVEDA was funded thanks to a Grant of Veneto Region in 2009. Samples were collected at Padua, Verona, Treviso, Vicenza and Portogruaro hospitals.

Netherlands, Department of Translational Neuroscience, The Rudolf Magnus Institute of Neuroscience, University Medical Center, Utrecht and Rintveld, Center for Eating Disorders, Altrecht in Zeist: Marek K. Brandys was supported by funding from the Marie Curie Research Training Network INTACT (Individually tailored stepped care for women with eating disorders; reference number: MRTN-CT-2006-035988). Martien Kas was supported by a ZonMW VIDI-Grant (91786327) from The Netherlands Organization for Scientific Research (NWO).

Norway, The National Institute of Public Health Twin Panel (NIPHTP): The NIPHTP was supported in part by grants from The Norwegian Research Council, The Norwegian Foundation for Health and Rehabilitation, The Norwegian Council for Mental Health and The European Commission under the program ‘Quality of Life and Management of the Living Resources’ of 5th Framework Program (no. QLG2-CT-2002-01254).

Poland, Poznan University of Medical Sciences (PUMS): PUMS study was sponsored by KBN scientific Grant no. PO5B 12823

Spain, Center for Genomic Regulation (CRG), Barcelona. Spanish Plan Nacional SAF2008-00357 (NOVADIS); the Generalitat de Catalunya AGAUR 2009 SGR-1502; the Instituto de Salud Carlos III (FIS/FEDER PI11/00733); and the European Commission 7th Framework Program, Project N. 261123 (GEUVADIS), and Project N. 262055 (ESGI).

Spain, Department of Psychiatry University Hospital of Bellvitge-IDIBELL, Barcelona: Financial support was received from Fondo de Investigación Sanitaria—FIS (PI11/210) and AGAUR (2009SGR1554). CIBER Fisiopatología de la Obesidad y Nutrición (CIBERobn) is an initiative of ISCIII.

Sweden, Karolinska Institutet, Stockholm: The Swedish Twin Registry is supported by the Swedish Department of Higher Education. The STR was supported by grants from the Ministry for Higher Education, the Swedish Research Council (M-2005-1112 and 2009-2298), GenomEUtwin (EU/QLRT-2001-01254; QLG2-CT-2002-01254), NIH Grant DK U01-066134, The Swedish Foundation for Strategic Research (SSF; ICA08-0047), the Swedish Heart-Lung Foundation, the Royal Swedish Academy of Science, and ENGAGE (within the European Union Seventh Framework Programme, HEALTH-F4-2007-201413).

United Kingdom, King’s College London: Financial support was received from the European Union (Framework-V Multicentre Research Grant, QLK1–1999-916), a Multicentre EU Marie Curie Research Training Network Grant, INTACT (MRTN-CT-2006-035988) and a Marie-Curie Intra-European Fellowship (FP-7-People-2009-IEF, No. 254774). Oliver Davis is supported by a Sir Henry Wellcome Fellowship from the Wellcome Trust (WT088984). Cathryn Lewis is partly supported by the National Institute for Health Research (NIHR) Biomedical Research Centre for Mental Health at South London and Maudsley NHS Foundation Trust and Institute of Psychiatry, King’s College London.

United States, McLean Hospital/Harvard Medical School, Cambridge, MA: The collection of DNA from participants at the McLean Hospital/Harvard Medical School site was supported in part by an investigator-initiated grant from Ortho-McNeil Janssen Scientific Affairs (principal investigator: Dr Hudson).

United States, University of North Carolina: Sample collection was funded by a grant from the Foundation of Hope, Raleigh, North Carolina. Sara Trace, Jin Szatkiewicz and Jessica Baker were funded by T32 MH076694 (PI: Bulik). Sara Trace was funded by a 2012–2015 Hilda and Preston Davis Foundation Postdoctoral Fellowship Program in Eating Disorders Research Award. Stephanie Zerwas was funded by a UNC BIRWCH award K12HD001441. The Clinical and Translational Science Award (CTSA) program at UNC-Chapel Hill provided additional assistance UL1TR000083.

United States, Vanderbilt University School of Medicine, Nashville TN, and the Kartini Clinic for Disordered Eating, Portland, OR: Cases were ascertained from the Kartini Clinic, Portland Oregon. Sample collection and processing was funded by a Bristol-Myers Squibb Freedom to Discover Unrestricted Metabolic Diseases Research grant to RDC.

# Full names and locations of all ethics committees that approved the study (documented informed consents can be obtained at each committee)

Canada, (Toronto), Centre for Addition and Mental Health Research Ethics Board

Czech Republic, (Prague), Charles University ethical committee VFN

Finland, (Helsinki), ethical committees associated with the University of Helsinki

Finland, Coordinating ethical committee of University of Kuopio

Finland, The hospital district of Helsinki and Uusimaa, The coordinating ethical committee

France, (Paris), ethical committees associated with the Faculty Paris VII Hôpital Louis Mourier

France, (Paris), Committee for the assessment of the protection of people in biomedical research, Pitié-Salpétrière hospital.

Germany, (Essen), ethical committees associated with the University of Essen

Greece, (Athens), ethical committees associated with the Athens University Medical School

Greece, (Athens), ethical committees associated with the Harokopio University

Italy, (Naples), University of Naples Ethic Committee

Italy, (Padua), ethical committees associated with the Veneto Region Padua Hospital

Netherlands, (Leidschendam), ethical committees associated with the Center for Eating Disorders Ursula

Netherlands, (Utrecht), ethical committees associated with the University of Utrecht

Netherlands, Patient-Related Scientific Research Review Committee

Netherlands, ethical committees associated with the Rudolf Magnus Institute, Department of Psychiatry, University Medical Center Utrecht, The Netherlands

Norway, (Oslo), University of Oslo Faculty of Medicine, Regional Committee for Medical Research Ethics, Southern Norway, Section A

Poland, (Poznan), Komisja Bioetycnza przy Uniwersytecie Medycznytm im. Karola Marcinkiwskiego w Poznan

Spain, (Barcelona), University Hospital of Bellvitge Comitè Ètic d'Investigació Clinica

Sweden, (Stockholm), Karolinkska Instutet Ethical Committee

UK, (London), Institute of Psychiatry Ethical Committee (Research)

UK, (London), The Joint South London and Maudsley and The Institute of Psychiatry NHS Research Ethics Committee

UK, (Leeds), ethical committees associated with the Leeds University/GSK/Dundee University

UK, HMDMC Sanger Institute

USA, (Harvard), McLean Hospital IRB

USA, (North Carolina), University of North Carolina Ethics Committee

USA, (Oregon,US), Oregon Health and Science University Institutional Review Board

USA, (Vanderbuilt), ethical committees associated with the Vanderbuilt University
