# Supplementary figures and images for: Machine learning derived risk prediction of anorexia nervosa
Source: BMC Med Genomics. 2016 Jan 20;9:4. doi: 10.1186/s12920-016-0165-x (PMC4721143; doi:10.1186/s12920-016-0165-x)

ROC plot

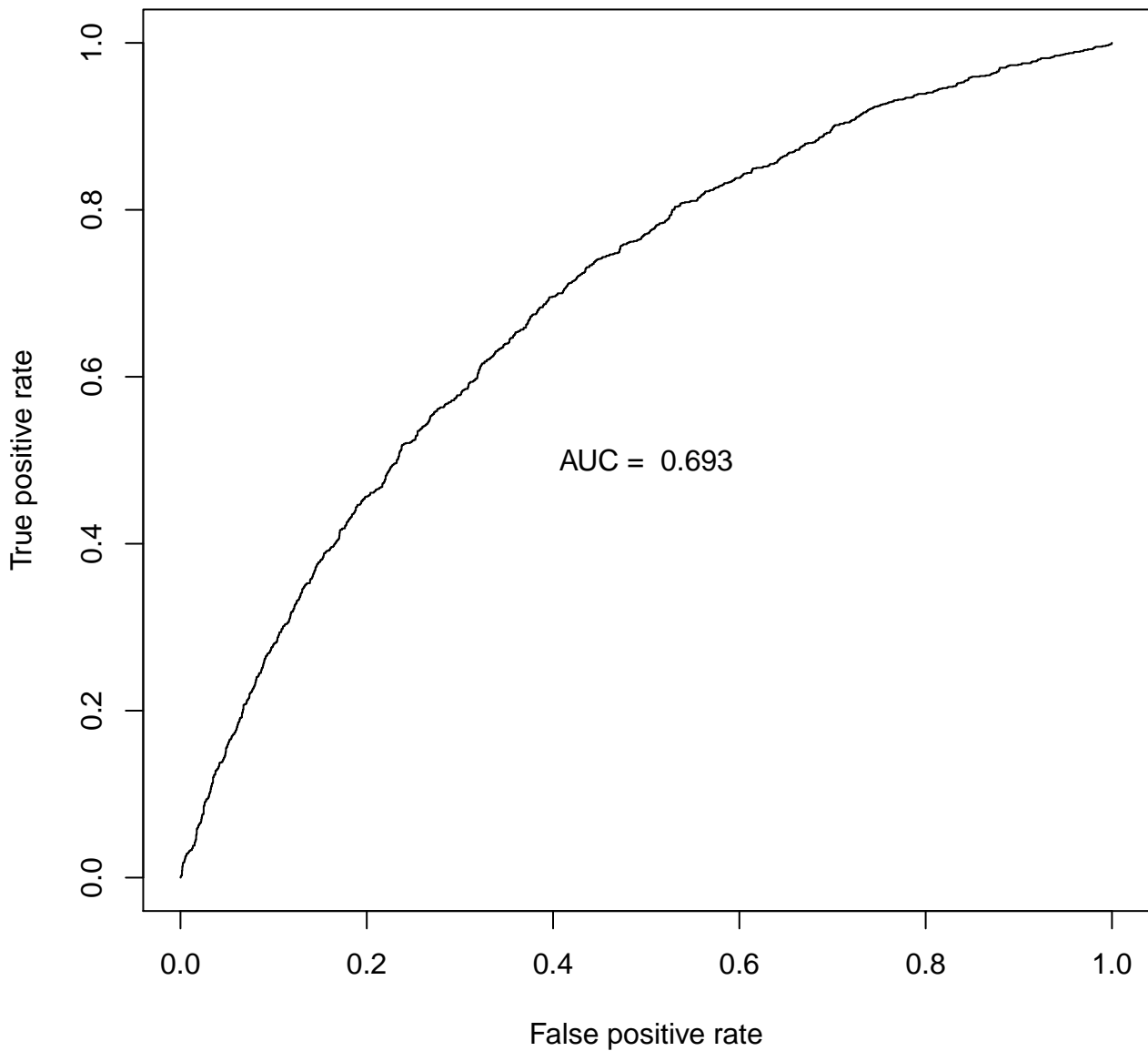

Supplement: Supplementary file 2 — ROC curve for linear regression model in shuffle 1. (PDF 17 kb) [file 12920_2016_165_MOESM2_ESM.pdf]
